# Myostatin Alteration in Pigs Enhances the Deposition of Long-Chain Unsaturated Fatty Acids in Subcutaneous Fat

**DOI:** 10.3390/foods11091286

**Published:** 2022-04-28

**Authors:** Yangli Pei, Yuxin Song, Zheng Feng, Hua Li, Yulian Mu, Saif ur Rehman, Qingyou Liu, Kui Li

**Affiliations:** 1 Guangdong Provincial Key Laboratory of Animal Molecular Design and Precise Breeding, Key Laboratory of Animal Molecular Design and Precise Breeding of Guangdong Higher Education Institutes, School of Life Science and Engineering, Foshan University, Foshan 528225, China; peiyangli@163.com (Y.P.); songyuxin3340@163.com (Y.S.); greatfz@126.com (Z.F.); okhuali@fosu.edu.cn (H.L.); saif_ali28@yahoo.com (S.u.R.); qyliu-gene@gxu.edu.cn (Q.L.); 2 Institute of Animal Sciences, Chinese Academy of Agricultural Sciences, Beijing 100193, China; mouyulian@caas.cn; 3 Agricultural Genomics Institute at Shenzhen, Chinese Academy of Agricultural Sciences, Shenzhen 518124, China

**Keywords:** myostatin, lipidomics, subcutaneous fat, long-chain unsaturated fatty acids, glycerolipids, sphingolipids, glycerophospholipids, pigs

## Abstract

In animals, myostatin (*MSTN*) is a negative regulator that inhibits muscle growth and repair. The decreased level of functional *MSTN* gene expression can change the amount and proportions of fats in pigs. In this study we determined the lipidomics of subcutaneous fat in *MSTN* single copy mutant pigs and evaluated the variations in lipid contents of the subcutaneous fat from *MSTN*^+/−^ and wild type Large White (LW) pigs via ultra-performance liquid chromatography–quadrupole/Orbitrap-mass spectrometry (MS). The results showed that the quantities of glycerolipids, sphingolipids, fatty acyls and glycerophospholipids were significantly changed, particularly, the molecular diacylglycerol in glycerolipids, long-chain unsaturated fatty acids, and ceramide non-hydroxy fatty acid-sphingosine in sphingolipids were remarkably increased in the *MSTN*^+/−^ group. Due to their positive bioavailability demonstrated by previous researches, these three lipids might be beneficial for human health. Further, the results of our study confirm that *MSTN* participates in the regulation of fat metabolism, and reduced expression of *MSTN* can ultimately influence the accumulation of lipid contents in the subcutaneous fat of pigs.

## 1. Introduction

Myostatin (*MSTN*) is a protein that inhibits the development of muscle tissue and its inactive form could interrupt the deposition of fat contents [1,2,3], thus farm animals with *MSTN* functional mutations or *MSTN* gene knockout might have higher percentage of lean meat and lower fat contents. Indeed, *MSTN* knockout pig breeds including Landrace pigs [4], Meishan [5], Large White/Landrace × Duroc [6], and Erhualian [7] have already been generated, and among them, *MSTN*-mutant (both *MSTN^+/−^* and *MSTN^−/−^*) Meishan, and Erhulian pigs are healthy, whereas *MSTN*-mutant Landrace pigs died within a week after birth, and three *MSTN^−/−^* Large White/Landrace × Duroc piglets died within 1 day. All of these *MSTN*-edited pigs had a reduced fat content with increased tenderness similar to *MSTN^−/−^* Meishan pigs, and a significantly lower body fat percentage about 3.27% was observed (*p* < 0.05) than that of wild type pigs [5]. Similarly, the backfat thickness of *MSTN^−/−^* Meishan pigs was also significantly lower (*p* < 0.05) [8]. Previously, the transcription activator-like effector nucleases (TALENs) were used to generate nonsense mutations (which act as stop codons) in the third exon of the *MSTN* gene of cells derived from both Meishan (MS) and Large White (LW) pigs which subsequently results in increased yields of lean meat in both LW (*MSTN*^+/−^) and MS (*MSTN*^−/−^ and *MSTN*^+/−^) pigs [9].

*MSTN* inhibition has also been investigated in other animals and is being employed as a potential strategy to decrease animal fat tissues. In transgenic mice, the *MSTN* gene was suppressed using a propeptide cDNA sequence resulting in the significant reduction of fat contents in the retroperitoneal, subcutaneous, and epididymal regions as compared to WT mice [2]. Additionally, the decreased level of visceral fat was also observed in adult mice once the *MSTN* was knocked down using siRNA [10]. Other studies have demonstrated that *MSTN* inhibition might be futile while inducing it for weight loss in obese mice [11]. Primarily, *MSTN* administration could inhibit adipogenesis in 3T3-L1 preadipocytes through down-regulating the expression of CCAAT/enhancer binding protein (C/EBP) β and peroxisome proliferator-activated receptor γ (PPARγ) [12], C/EBPα, and lipid metabolism-related genes such as diacylglycerol O-acyltransferase (DGAT), glycerol-3-phosphate dehydro-genase (GPDH), adipose triglyceride lipase (ATGL), hormone-sensitive lipase (HSL), and acylCoA synthetase long-chain family member 1 (ACS1) [13]. Moreover, *MSTN* supplementation to adipogenic differentiation medium suppressed the adipogenesis in porcine and bovine preadipocytes [14,15], while it can promote adipogenesis through driving the pluripotent stem cells into a particular state [16]. Similarly, in animals, the *MSTN* gene inhibition or deletion can decrease fat contents and increase the muscle mass. Furthermore, the reduced fat mass is particularly associated with the inhibition of the *MSTN* in muscle tissue (though not adipose tissue), whereas the *MSTN* is helpful in regulating fatty acids metabolism through MEF2C/miR222/SCD5 cascade, and further affect the fat deposition and TG biosynthesis [8]. Therefore, *MSTN* plays a substantial role in controlling or regulating both myogenesis and adipogenesis. Fat is one of the important macronutrients of our daily diets, which also enhances the flavor and aroma of food. Our bodies require small amounts of essential fatty acids from food on a daily basis to construct cell membranes and to produce many vital hormones including testosterone, progesterone, prostaglandins, and estrogen. However, obesity contributes to many chronic diseases and can be caused by excessive fat intake [17]. Thus, the bioavailability of dietary fat or lipids with anti-obesity properties have become topics of great interest for researchers in recent decades [18]. Moreover, the *MSTN* inhibition may contribute to lower the amount of adipose tissue in animals. However, it is yet not clear whether the functional inactivation of the *MSTN* can alter the composition of fat which subsequently affects the properties of dietary fat. Therefore, in current study we employed untargeted metabolomics based on ultra-performance liquid chromatography–quadrupole/Orbitrap-mass spectrometry (LC-MS) to explore the phenotypic changes in lipid composition of subcutaneous fat from *MSTN*-edited Large White pigs.

## 2. Materials and Methods

### 2.1. Animals

All the animal experiments were approved by the Animal Welfare and Research Ethics Committee at the Institute of Animal Sciences, Chinese Academy of Agricultural Sciences (IAS2018-10). The *MSTN*-edited Large White (LW) pigs were generated using TALEN [9]. The *MSTN* gene carried by this pig is prematurely produced by a termination codon in the 3rd exon due to a base mutation (A/G), following an 11 bp deletion [9]. All the pigs were kept under same conditions with free access to commercial pig diets (Tianjin Taikang feed mill, Tianjin, China) and water.

### 2.2. Sampling

Pigs were categorized into two groups: wild type (WT) and heterozygous mutant (*MSTN*^+/−^) pigs. In total, eight 8-month-old pigs (five boars that were not castrated and three non-pregnant sows) were included in each group. The average body weight (ABW) of *MSTN*^+/−^ and WT LW pigs were 114.44 ± 3.68 kg and 115.37 ± 3.65 kg, respectively. All the animals were reared under the same feeding conditions. Pigs were sacrificed in a slaughter-house by electric shock and incision of the carotid artery. The dorsal subcutaneous adipose tissues above the longissimus dorsii were removed, and immediately soaked in liquid nitrogen for rapid freezing, and then stored at −80 °C.

### 2.3. Histologic Analysis

Adipose tissues morphology was examined by using hematoxylin–eosin (HE) staining. Through a series of graded ethanol solutions, 5 μm-thick paraffin sections were de-waxed and re-hydrated. Then, water was used to wash the adipose tissues sections and those hydrated sections were treated with 5% hematoxylin solution for 5 min, and then tap water was used to rinse the sections. After that, the adipose tissue sections were treated with hematoxylin differentiation solution, and tap water was used to rinse the section again. Following that, adipose tissue sections were stained for 5 min with a 0.5% eosin solution. Finally, the stained sections were examined under an LEICA DMi8 microscope [19]. ImageJ was used to measure the total adipocyte size. In order to calculate the adipocyte size, we calculated the ratio of total adipocyte size/number of adipocytes, and the imperfect cells were removed manually.

### 2.4. Lipid Extraction

Subcutaneous fat samples were thawed using ice, and isopropanol (IPA) was used to obtain the lipids. After adding precooled IPA (120 μL) to the samples, the vortexing was performed for 1 minute and the samples were then incubated for 10 min at room temperature. The resulting mixture was stored overnight at −20 °C. The supernatants were centrifuged for 20 min at 4000 g, after that, each sample was diluted to 1:10 using IPA/acetonitrile (ACN)/H_2_O (2:1:1, *v*:*v*:*v*) and the samples were kept at −80 °C for prior analysis. Furthermore, a 10 μL extraction mixture from each sample was used to prepare the QC samples.

### 2.5. Lipidomics Study

All the lipid samples were evaluated using LC-MS-based ultra-performance liquid chromatography (UPLC) system (SCIEX, Warrington, UK) and a high-resolution tandem mass spectrometer TripleTOF5600plus (SCIEX, UK). Some metabolic ions were positively charged, whereas others were negatively charged so, to detect metabolic ions as many as possible a quadrupole time-of-flight (Q-TOF) mass spectrometer was operated separately in both electrospray ionization positive (ESI+) and negative (ESI−) modes. The electrospray voltages were 5000 V in the positive ion mode and 4500 V in the negative ion mode.

The XCMS software was used to preprocess the LC-MS collected data and then we converted the raw data files to mzXML and process those using R’s XCMS, CAMERA, and metaX tools. Based on retention time and *m*/*z* information, the ions were identified. We recorded the intensity of each peak and generated a three-dimensional matrix containing sample names (observations), arbitrarily assigned peak indices (retention time-*m*/*z* pairs), and ion intensity information (variables). To identify level-one and level-two (MS2) metabolites, the public databases (Kyoto Encyclopedia of Genes and Genomes, KEGG, and Human Metabolome Database, HMDB), and in-house databases which contain 8360 samples were used for identification and annotation.

### 2.6. Data Analysis

A *t*-test was used to identify the differences in the concentrations of metabolites between WT and *MSTN*^+/−^ pigs. For identified metabolites, Principal Component Analysis (PCA) was performed using the R function prcomp and 2D PCA plot with R package rgl. Each point in the PCA figures represented a sample. Separation and aggregation trends of samples in the score chart reflected the similarity and differences among all samples. The R package metaX was used to perform a partial least squares-discriminant analysis (PLS-DA) to evaluate the variables between groups. Furthermore, a 200-permutation test was executed in order to validate the PLS-DA model by describing the R^2^Y and Q^2^ values. Volcano plots were used to evaluate the base R volcano plot function. Additionally, the variable important in projection (VIP) value was analyzed from the OPLS-DA model and VIP ≥ 1, *p* < 0.05 and fold changes ≥2 or ≤−2 values were considered as a statistical criterion to determine the differentially expressed lipid metabolites.

### 2.7. Pathway Analysis

In MetabolAnalyst 5.0 (https://www.metaboanalyst.ca/, accessed on 15 June 2021), Metabolomics Pathway Analysis (MetPA) was used to analyze the metabolic pathways of differentially expressed lipid metabolites. The pathway topology analysis was also used to determine the *p* value for pathway enrichment analysis and pathway impact values.

## 3. Results

### 3.1. Basic Information on Experimental Pigs and LC-MS-Based Untargeted Lipidomics Results

**Figure 1:**
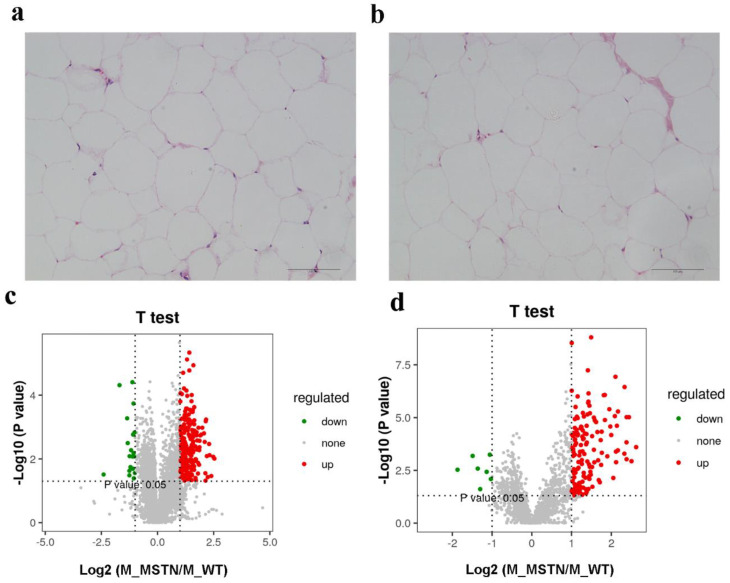
The decreased expression of *MSTN* altered the lipid content of subcutaneous fat. (**a**) HE staining of adipose tissue from *MSTN*^+/−^ pigs; (**b**) HE staining of adipose tissue from WT pigs; (**c**) Volcano plot of lipid metabolites in positive ion mode; (**d**) Volcano plot of lipid metabolites in negative ion mode.

The *MSTN^+/−^* LW pigs showed significantly reduced fat contents than that of WT LW pigs (5.108% ± 0.5356% vs. 10.36% ± 1.165%, *p* < 0.01) [9]. Histological examination of the subcutaneous fat sections exhibited that the adipocytes in *MSTN*^+/−^ and WT adipose tissues were both round or oval with individual well-circumscribed cell borders (Figure 1a,b), and the adipocytes size calculated by ImageJ did not significantly differ between two groups (*p* > 0.05). These results revealed that there was no change in the adipocyte morphology and distribution between *MSTN*^+/−^ and WT pigs. Furthermore, we used LC-MS-based untargeted lipidomics to examine the subcutaneous fat of *MSTN*^+/−^ (n = 8) and WT LW pigs (n = 8) and evaluated the variable lipids composition in subcutaneous fat as a response to *MSTN*-alteration. In total, we detected 5259 metabolites in positive ion mode, of which 2959 were annotated, with 410 matched to in-house databases (MS2). Moreover, 1756 metabolites were perceived in negative anion mode, of which 844 were annotated, with 44 matched to in-house databases (MS2). We also identified 454 MS2 metabolites in negative and positive ion modes (Table 1). Additionally, the volcano plot presentation showed that most of the metabolites detected from both positive and negative ion modes were upregulated in the subcutaneous fat of *MSTN*-edited pigs (Figure 1c,d).

### 3.2. PCA and PLS-DA Analysis of Lipid Metabolism Profiles

**Figure 2:**
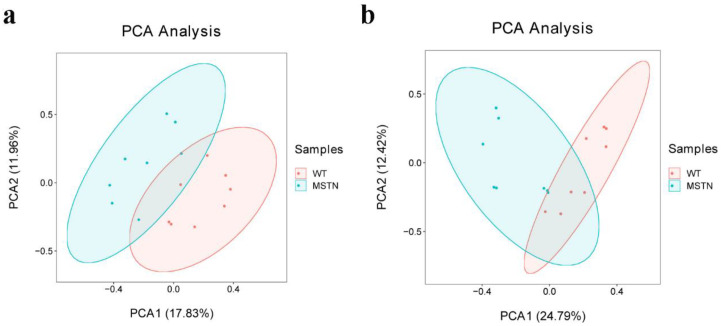
PCA scores of the detected compounds in the two groups. (**a**) PCA scatter plot of differentially expressed lipid metabolites in positive ion mode; (**b**) PCA scatter plot of differentially expressed lipid metabolites in negative ion mode.

**Figure 3:**
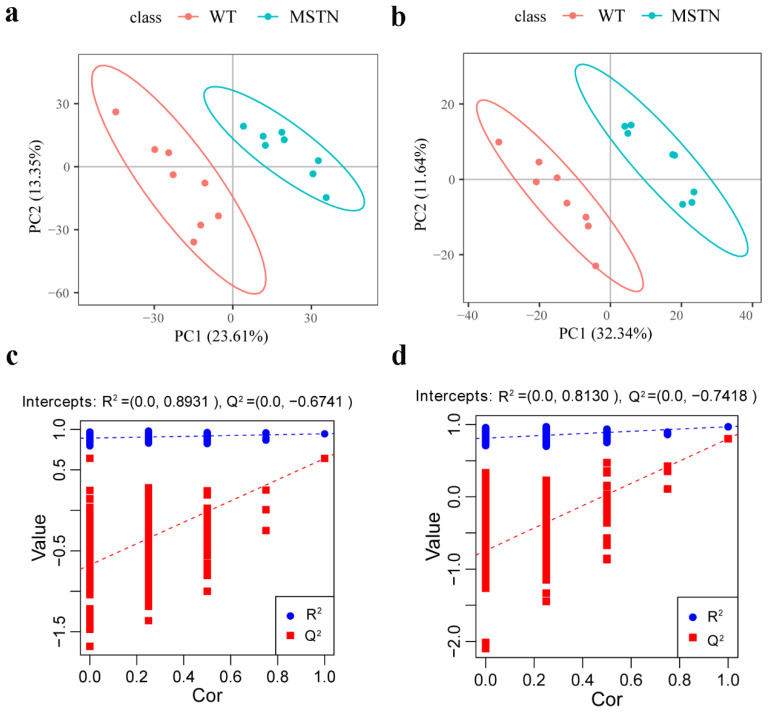
PLS-DA of lipid metabolite profiling data for comparison of *MSTN*^+/−^ and WT pigs. (**a**) PLS-DA score plots of WT and *MSTN*-edited pigs based on the extracted spectral data in positive ion mode; (**b**) PLS-DA score plots of WT and *MSTN*-edited pigs based on extracted spectral data in negative ion mode; (**c**) Permutation plot of PLS-DA based on the extracted spectral data in positive ion mode. R^2^Y = 0.8931 and Q^2^ = 0.6741; (**d**) Permutation plot of PLS-DA based on the extracted spectral data in negative ion mode. R^2^Y = 0.8130 and Q^2^ = 0.7418.

In order to compare the metabolic lipid profiling of subcutaneous fat between *MSTN^+/−^* and WT pigs, we conducted Principal Component Analysis (PCA), and results are shown in Figure 2a,b. In unsupervised mode, the samples of the two groups exhibited obvious clustering. In addition, we also used a partial least squares discriminant analysis (PLS-DA) to determine the distinct lipid metabolites in subcutaneous fat of the *MSTN^+/−^* and WT pigs. A significant difference in the subcutaneous fat of two genotypes (Figure 3a,b) was observed, which indicated that the *MSTN* alteration could produce significant changes in biochemistry of the subcutaneous fat. Further, we validated our PLS-DA model, and the values of R^2^Y and Q^2^, were verified by a 200-times permutation test. Notably, the R^2^Y and Q^2^ support the fitness and prediction power of PLS-DA model, respectively, and the values of R^2^Y and Q^2^ > 0.5 are considered as the best fit for model verification. The detected values of R^2^Y and Q^2^ for our model were 0.8931 and 0.6741 in positive ion mode, and 0.8130 and 0.7418 in negative ion mode, respectively (Figure 3c,d). The results indicated that there is no over-fitting in this model, and the analysis of differentially expressed metabolites is accurate.

### 3.3. Screening the Differential Lipid Metabolites

**Figure 4:**
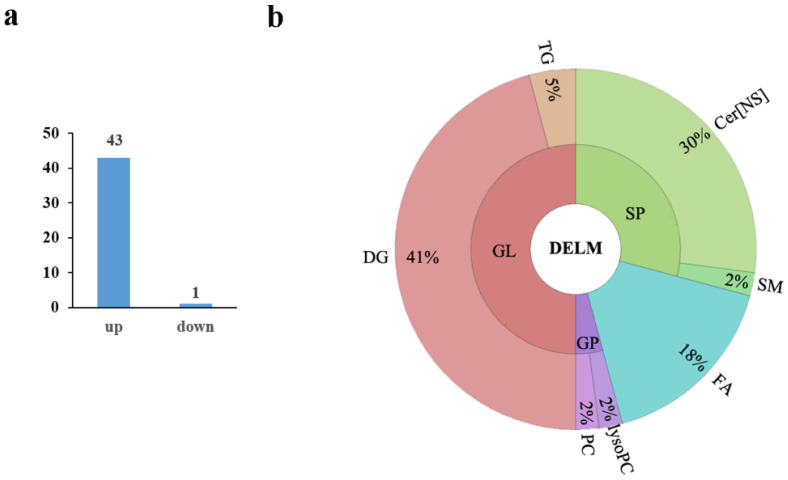
Differentially expressed lipid metabolites in the *MSTN*^+/−^ versus the WT pigs. (**a**) Of the 44 differentially expressed metabolites, 43 were upregulated and 1 was downregulated; (**b**) The differential metabolites included 24 glycerolipids (GL), 14 sphingolipids (SP), 8 fatty acyls (FA), and 2 glycerophospholipids (GP). There were 22 diacylglycerol (DG) and 2 triacylglycerol (TG) molecules in the 24 differentially expressed glycerolipids. There were 13 ceramide non-hydroxy fatty acid-sphingosine (Cer[NS]) and 1 sphingomyelin (SM) in differential sphingolipids.

**Figure 5:**
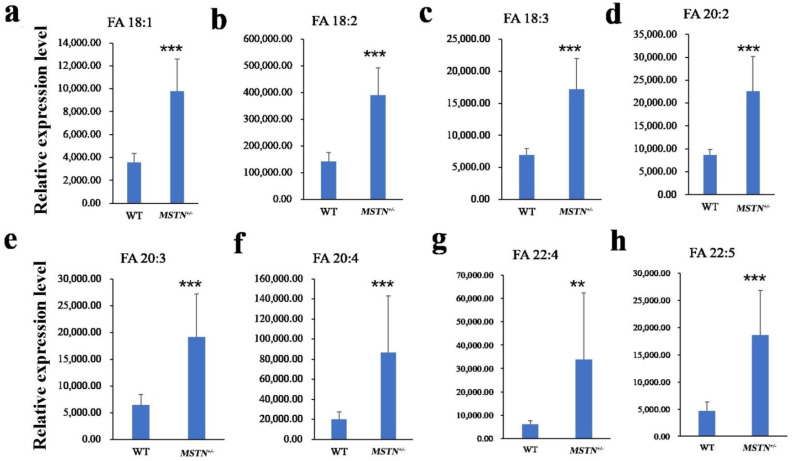
Differentially expressed long-chain unsaturated fatty acids in the *MSTN*^+/−^ versus the WT pigs. (**a**–**h**) Relative expression level of FA 18:1 (**a**), FA 18:2 (**b**), FA 18:3 (**c**), FA 20:2 (**d**), FA 20:3 (**e**), FA 20:4 (**f**), FA 22:4 (**g**), FA 22:5 (**h**) was significantly different between *MSTN*^+/−^ and WT pigs. Note: ** *p* < 0.01, *** *p* < 0.001.

The Q-value obtained by Benjamini–Hochberg (BH) correction using univariate fold-change analysis, the *t*-test, and the VIP value obtained by PLS-DA were analyzed by multivariate statistical analysis and were used to screen the differentially expressed lipid metabolites. We have identified a total of 44 differentially expressed lipid metabolites (DELM) between *MSTN*-edited and WT LW pigs based on relative abundance and VIP values, of which 43 were upregulated and only 1 was downregulated (Figure 4a). These differentially expressed metabolites included 20 glycerolipids (GL), 14 sphingolipids (SP), 8 fatty acyls (FA), and 2 glycerophospholipids (GP) as shown in Figure 4b. The 8 differentially expressed FAs were all long-chain unsaturated fatty acids (molecules with >12 carbon atoms) (Table 2 and Figure 5). From 20 differentially expressed glycerolipids, 18 were diacylglycerols (DG) and 2 triacylglycerols (TG), and all the 18 diacylglycerols were up-regulated, while, from 2 triacylglycerols, the TG 42:4 was upregulated whereas the other TG 50:0 was downregulated. All the 22 altered DGs contained polyunsaturated bonds (Table 3). Of the 14 differentially expressed sphingolipids, there were 13 ceramide non-hydroxy fatty acid-sphingosines (Cer[NS]) and only 1 was sphingomyelin (SM). All of the 14 differentially expressed sphingolipids were upregulated in the *MSTN*-edited group (Table 4). Similarly, of the 2 altered GPs, there was 1 lysophophatidylcholine (lysoPC) and 1 phosphatidylcholine (PC, Table S1).

### 3.4. Pathway Analysis of the Differentially Expressed Lipids

**Figure 6:**
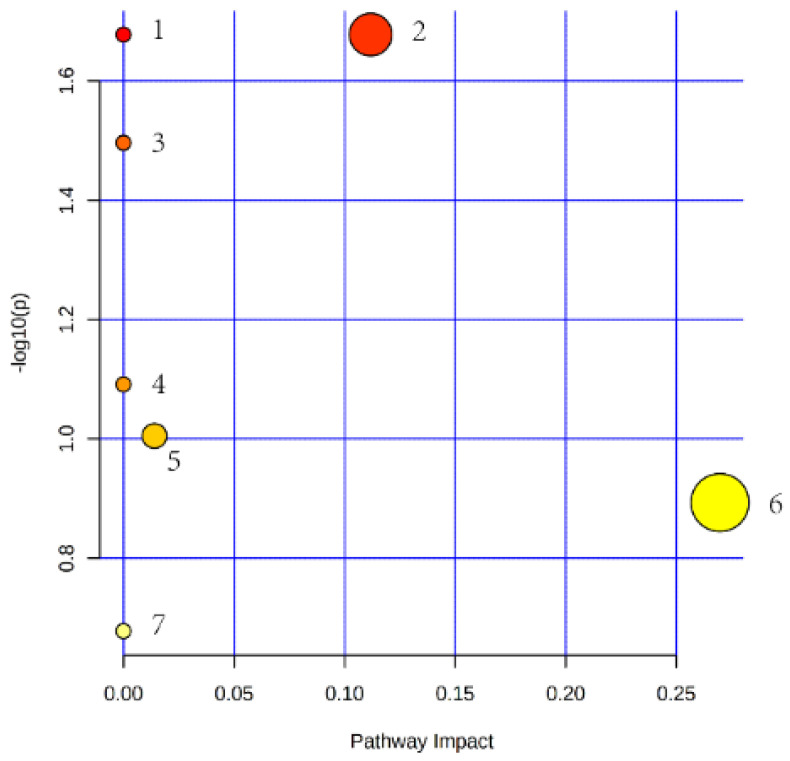
Lipid metabolic pathway analysis of the identified differential lipid species. (The circle size and x-axis represent the pathway impact value, and the circle color and y-axis is the -log (*p*-value) obtained from pathway enrichment analysis. 1: pathway of biosynthesis of unsaturated fatty acids; 2: pathway of glycerophospholipid metabolism; 3: pathway of linoleic acid metabolism; 4: pathway of alpha-linolenic acid metabolism; 5: pathway of glycerolipid metabolism; 6: pathway of sphingolipid metabolism; 7: pathway of arachidonic acid metabolism.)

A total of seven pathways, including biosynthesis of unsaturated fatty acids, glycerophospholipid metabolism, linoleic acid metabolism, alpha-linolenic acid metabolism, glycerolipid metabolism, sphingolipid metabolism, and arachidonic acid metabolism were accessed as potential associated pathways related to the differentially expressed lipid. The pathway impact analysis of these seven pathways is presented in Figure 6.

## 4. Discussion

Pig meat contributes 40% of the global red meat consumption. Superfluous fat intake is thought to be associated with an increased risk of many diseases, such as ovarian cancer [20], Barrett’s carcinogenesis [21] and gestational diabetes mellitus [22]. So, lower fat content meat with healthier lipids has an increasing importance in the pig breeding goal because of their invaluable impact on consumer acceptance. Both single base or double site mutation in *MSTN* gene can reduce the fat content of pigs. Further, the growth of single base mutation pigs is normal and the reproductive organs, and digestive organs have no difference from wild type pigs [9,23]. In the present study, we investigated the effects of *MSTN* single allele editing on the lipid composition of the subcutaneous fat of *MSTN*-edited LW pigs. The results exhibited no significant differences in subcutaneous adipose, and differential lipid metabolites including glycerolipids, sphingolipids, fatty acyls, and glycerophospholipids.

The loss of *MSTN* function in pig, rabbit, cattle, and sheep, etc., results in a reduction of total fat mass [24]. Here, we found insignificant changes in adipocyte morphology and distribution between *MSTN*^+/−^ and WT pigs. Moreover, in *MSTN^−/−^* mice, the adipocyte size of inguinal fat pads appeared smaller until day 62 [25]. Whereas, in both male and female *MSTN^−/−^* mice, the size of adipocytes in white adipose tissue was smaller than that of WT mice, but, smaller size of gonadal fat pads and the intrascapular brown fat was only observed in female *MSTN^−/−^* mice [26]. Although all the tissues were taken from *MSTN^−/−^* mice, but the adipocyte size of tissue from different genders and distinct tissues showed dissimilar results. In addition, it was also reported that *MSTN* inhibited intramuscular preadipocyte differentiation in a dose-dependent manner in 3T3-L1 cells [15]. Our results are inconsistent with earlier reports, possibly due to different genotypes, different species, and the dose-dependent impact of *MSTN* on fat cells.

The down-regulation of the *MSTN* gene promoted the accumulation of long-chain unsaturated fatty acids, especially the monounsaturated fatty acids including oleic acid and polyunsaturated fatty acids, such as linoleic acid and arachidonic acid. Fatty acids play an important role in the human diet and subsequently prevent various types of diseases, such as polyunsaturated fatty acid (PUFA), which might reduce the risk factors of end-stage renal disease or failure through a variety of mechanisms in populations with a high burden of kidney disease [27]. Additionally, PUFA can impart a protective role in patients with cardiovascular and cerebrovascular diseases [28]. Similarly, long-chain PUFAs are indeed inducive for the people suffering from obesity and osteoporosis [29]. Beside this, oleic acid (FA 18:1) insures against cancer, insulin resistance, and type 2 diabetes mellitus [30,31]. Omega3 PUFAs, a conjugant of linoleic acid (FA 18:2), and the linolenic acid, also possess inhibitory properties for cancer development [30]. A previous study in rats has demonstrated that fructose plus an oleic, linoleic, and α-linolenic fatty acids-enriched diet could reduce body fat [32]. Linoleic acid is a precursor of arachidonic acid (FA 20:4), which is essential for the proper development of the immune, skeletal muscle, and nervous systems particularly, for optimal brain and cognitive performance [33]. Earlier, the results of our study in *MSTN*-edited pigs also revealed higher contents of PUFA in longissimus dorsi muscles [9]. Moreover, transcriptomic analyses of the *MSTN^−/−^* knockout mice also disclosed several sub-networks involved in fatty acid metabolism which were significantly enriched at d35 [34]. Furthermore, comparative proteomic and phosphoproteomic analyses in *MSTN^−/−^* cattle illustrated that *MSTN* knockout could enhance the activity of many key enzymes involved in fatty acid β-oxidation and glycolysis processes in gluteus muscle tissues [35]. Besides, *MSTN* was also demonstrated to regulate the desaturation of fatty acids through MEF2C/miR222/SCD5 cascade in subcutaneous fat of *MSTN*-knockout Meishan pigs [8]. Therefore, *MSTN* is involved in fatty acid metabolism and affects the content of fatty acids in subcutaneous fat.

The DG concentration in subcutaneous adipose tissue increased in *MSTN^+/−^* pigs. In two human population groups it was observed that the human group who took diacylglycerol (mainly 1,3-diacylglycerol) had a lower level of postprandial triglyceridemia than that of the human group who consumed triacylglycerol [36]. Dietary diacylglycerol has reduced the hepatic fat accumulation caused by high-fat diets in rats, as well as accumulated abdominal fat and body weight in Japanese males [36,37]. A clinical double-blind and random trial demonstrated that diacylglycerol oil-supplemented foods is helpful in downsizing body fat and also accelerates weight loss in men and women who were considered overweight (waist circumference ≥ 90 cm and waist circumference ≥ 87 cm, respectively) [36]. Microencapsulated duck DG intake was found to be safe and acted as a functional ingredient for decreasing body weight, lowering obesity, and inducing the metabolism of lipids in the viscera of hyperlipidemic Wistar rats [18]. Furthermore, the DG positively affected the growth performance and nutrient digestibility in Ross broiler chicks [38]. Thus, the DG consumption cannot only be helpful in reducing the postprandial TG levels in serum, suppress abdominal and visceral fat accumulation, lessen obesity, promote growth performances, and nutrient digestibility, but can also assist in the treatment of clinical metabolic diseases. The glycerol phosphate pathway is the main synthesis pathway of triacylglycerol in which DG acyltransferase esterified the DG and converted it into TG [39]. It has also been reported that the concentration of TG in adipocytes was decreased up to 46% after *MSTN* treatment [40]. The TG proportion in the subcutaneous fat tissue of *MSTN*^+/−^ and *MSTN*^−/−^ pigs was decreased [8], while in *MSTN*^−/−^ pigs the expression level of the genes involved in lipid synthesis were also found to be downregulated in the adipose tissue [41]. Here we perceived that the decreased DG contents were probably associated with the loss of *MSTN* function which prevents the formation of TG from DG.

The results of our study showed that the contents of Cer[NS] were increased in subcutaneous fat of the *MSTN*^+/−^ pigs. Fatty acid and sphingosine constitute to form ceramides (Cer) which are signalling molecules involved in the regulation of inflammation, insulin resistance related to obesity, decreased oxidation of fatty acids, and liver steatosis [42]. Cer, which consist of about half of the intercellular lipids, are important for enhancing the skin ability to act as a barrier, inhibiting melanin production [43] and moisturizing the skin [44]. Cer are a vital entity owing to their functional involvement in different cellular activities or biological functions. This opens up ways to access their ability to treat sensitive and dry skin and their possible role in healthy foods and cosmetics. There are several examples of these products being produced at a commercial level and sold, and the market for such goods is constantly growing (e.g., Kao Men’s Bioré Double Moisture Cream [Kao Corporation, Tokyo, Japan], L’Actua Deep Harmonize Cream [Ajinomoto Health Supply, Tokyo, Japan, Eucerin Smoothing Repair Dry Skin Lotion [Beiersdorf, Inc, Hamburg, Germany], Eucerin Eczema Relief Body Crème, CeraVe Moisturizing Lotion [Valeant Pharmaceuticals International, Inc, Bridgewater, NJ], CeraVe Suncare Sunscreen Face SPF 30) [44]. Cer[NS], contains a non-hydroxyl acyl and sphingosine and is an important ceramide. *Saccharomyces cerevisiae* have already been used to produce human Cer[NS] [45]. Further, the Cer_NS were also isolated from porcine hard palates by using preparative thin-layer chromatography [46]. However, the bioavailability of Cer[NS] from *S. cerevisiae* and the stratum corneum from the porcine hard palate has not yet been verified, while the biological characteristics of naturally derived Cer are similar to the Cer found in the human skin [47]. According to our results, *MSTN* plays a significant role in increasing the quantity of Cer[NS] in adipose tissue. The subcutaneous fat of our *MSTN*-edited pigs was enriched with Cer[NS], which cannot only be used as the source material for the extraction of Cer[NS], but can also provide a more direct source of Cer[NS] for consumers.

The pathway analysis presented a total of seven pathways significantly associated to the differentially expressed lipids. The results of transcriptome data from longissimus dorsi muscles of *MSTN*^−/−^ goats presented a number of genes, which were involved in fatty acid metabolism and significantly changed the biosynthesis of unsaturated fatty acids [48]. Previously it has also been stated that differentially expressed metabolites in the jejunum of *MSTN*^+^^/−^_and WT pigs were involved in sphingolipid metabolism, while half of the differently enriched metabolites in the cecum were lipids, which were mainly involved in glycerophospholipid and pyrimidine metabolism [49]. Thus, previously reported findings and our data suggest that *MSTN* could participate in the regulation of lipid metabolism in multiple species and organs.

## 5. Conclusions

Taken together, our study presented that molecular diacylglycerol in glycerolipids, long-chain unsaturated fatty acids, and Cer[NS] in sphingolipids were distinctly increased in the *MSTN*-edited group as compared to the WT group. The results of our study, along with previously reported studies, state that these lipid species have positive biological activity and excellent bioavailability, which may contribute to promote human health. Further, we confirmed that *MSTN* regulates the lipid metabolism of subcutaneous fat and can change the content of many lipids. Our study provides a comprehensive insight into the unique nutritional value of subcutaneous fat, which is one of the most common concerns in domestic animal breeding.

## Figures and Tables

**Table 1:** LC-MS-based untargeted lipidomics identification results statistics.

Mode	All Feature	All Annotated	MS2	HMDB	KEGG
pos	5259	2959	410	2551	1081
neg	1756	844	44	679	375

Note: pos, positive mode. neg, negative mode. MS2, the metabolites that matched with level-two fragment ions in in-house database.

**Table 2:** Significantly differently expressed fatty acyls in the *MSTN*^+/−^ versus the WT pigs.

MS2 Metabolites	WT	*MSTN*	FC (*MSTN*/WT)	*t*.Test_*p*.Value	VIP	Regulated	MS2class
FA 18:1	3546.793	9773.901481	2.7557	<0.0001	2.0193	up	FA
FA 18:2	143,397.6	391,532.6511	2.7304	<0.0001	2.0036	up	FA
FA 18:3	6932.366	17,210.03558	2.4826	<0.0001	1.9423	up	FA
FA 20:2	8642.403	22,567.74914	2.6113	0.0002	1.8699	up	FA
FA 20:3	6461.461	19,078.51236	2.9527	0.0003	1.9203	up	FA
FA 20:4	19,995.97	86,817.52091	4.3418	0.0004	2.1756	up	FA
FA 22:4	5978.082	34,021.06063	5.6910	0.0012	2.2632	up	FA
FA 22:5	4734.286	18,623.7508	3.9338	<0.0001	2.2632	up	FA

Notes: FA, fatty acids. WT, wild type group. *MSTN*, *MSTN*^+/−^ group. FC, fold change. VIP, variable important in projection.

**Table 3:** Significantly differently expressed glycerolipids in the *MSTN*^+/−^ versus the WT pigs.

MS2 Metabolites	WT	*MSTN*	FC (*MSTN*/WT)	*t*.Test_*p*.Value	VIP	Regulated	MS2class
DG 30:2; DG(12:0/18:2)	8560.459	20,125.29867	2.3510	0.0055	2.1145	up	DG
DG 33:0; DG(16:0/17:0)	5994.942	12,379.94687	2.0651	0.0033	1.8401	up	DG
DG 33:2; DG(15:0/18:2)	5044.778	12,492.03431	2.4762	0.0054	1.9723	up	DG
DG 34:2; DG(16:0/18:2)	170,198.4	355,028.0342	2.0860	0.0060	1.8450	up	DG
DG 34:3; DG(16:1/18:2)	103,739.4	233,561.7196	2.2514	0.0040	1.9747	up	DG
DG 34:4; DG(16:1/18:3)	6334.194	13,508.78926	2.1327	0.0005	2.1073	up	DG
DG 35:0; DG(17:0/18:0)	9121.664	24,772.23257	2.7158	0.0105	2.2335	up	DG
DG 35:1; DG(17:0/18:1)	14,536.48	34,174.0373	2.3509	0.0003	2.1624	up	DG
DG 35:2; DG(17:0/18:2)	22,347.37	49,492.0652	2.2147	0.0058	1.8518	up	DG
DG 35:3; DG(17:1/18:2)	8888.985	18,309.88358	2.0598	0.0152	1.6628	up	DG
DG 36:2; DG(18:1/18:1)	62,498.74	151,512.7417	2.4243	0.0038	2.0144	up	DG
DG 36:3; DG(18:1/18:2)	34,011.48	115,883.6619	3.4072	0.0026	2.3690	up	DG
DG 36:4; DG(18:2/18:2)	32,631.76	1,002,795.866	3.8613	0.0017	2.5261	up	DG
DG 36:5; DG(18:2/18:3)	32,631.76	115,389.4501	3.5361	0.0010	2.5030	up	DG
DG 37:3; DG(19:1/18:2)	4571.692	10,533.324	2.3040	0.0012	2.1446	up	DG
DG 38:4; DG(19:2/19:2)	18,561.4	58,653.825	3.1600	0.0016	2.3852	up	DG
DG 40:4; DG(18:0/22:4)	5632.562	12,793.07064	2.2713	0.0023	2.0061	up	DG
DG 40:5; DG(18:1/22:4)	10,866.66	22,187.87537	2.0418	0.0111	1.9783	up	DG
TG 42:4; TG(12:1/12:1/18:2)	7388.643	15,922.36588	2.1550	0.0190	1.9034	up	TG
TG 50:0; TG(16:0/16:0/18:0)	349,899.6	169,207.3744	0.4836	0.0084	1.9507	down	TG

Notes: DG, diacylglycerol. TG, triacylglycerol. WT, wild type group. *MSTN*, *MSTN*^+/−^ group. FC, fold change. VIP, variable important in projection.

**Table 4:** Significantly differently expressed sphingolipids in the *MSTN*^+/−^ versus the WT pigs.

MS2 Metabolites	WT	*MSTN*	FC (*MSTN*/WT)	*t*.Test_*p*.Value	VIP	Regulated	MS2class
Cer[NS] 32:1; Cer[NS](d18:1/14:0)	3861.602	9693.892561	2.5103	0.0282	2.0953	up	Cer_NS
Cer[NS] 33:1; Cer[NS](d17:1/16:0)	1068.501	2993.02488	2.8011	0.0044	2.1338	up	Cer_NS
Cer[NS] 34:1; Cer[NS](d18:1/16:0)	31,982.4	100,204.3959	3.1331	0.0117	2.3630	up	Cer_NS
Cer[NS] 34:2; Cer[NS](d18:1/16:1)	4002.504	17,169.607	4.2897	0.0029	2.7466	up	Cer_NS
Cer[NS] 35:1; Cer[NS](d18:1/17:0)	1267.395	5045.999952	3.9814	0.0011	2.8133	up	Cer_NS
Cer[NS] 36:1; Cer[NS](d18:1/18:0)	14,143.47	39,845.93159	2.8173	0.0184	2.2561	up	Cer_NS
Cer[NS] 36:2; Cer[NS](d18:2/18:0)	2028.951	6282.032455	3.0962	0.0170	2.0525	up	Cer_NS
Cer[NS] 36:4; Cer[NS](d17:3/19:1)	57,570.47	142,091.5412	2.4681	0.0200	1.9485	up	Cer_NS
Cer[NS] 38:1; Cer[NS](d18:1/20:0)	14,266.6	41,073.31911	2.8797	0.0186	2.1327	up	Cer_NS
Cer[NS] 38:2; Cer[NS](d18:2/20:0)	1844.387	6254.25343	3.3910	0.0051	2.6342	up	Cer_NS
Cer[NS] 42:1; Cer[NS](d18:1/24:0)	16,619.03	43,973.47298	2.6460	0.0412	1.9252	up	Cer_NS
Cer[NS] 42:2; Cer[NS](d18:1/24:1)	20,262.9	48,511.80825	2.3941	0.0399	1.9521	up	Cer_NS
Cer[NS] 42:3; Cer[NS](d18:1/24:2)	10,174.64	26,046.28408	2.5599	0.0162	2.1515	up	Cer_NS
SM 36:1; SM(d14:0/22:1)	14,790.72	30,015.73873	2.0294	0.0172	1.8125	up	SM

Notes: Cer[NS], ceramide non-hydroxy fatty acid-sphingosines. SM, sphingomyelin. WT, wild type group. *MSTN*, *MSTN*^+/−^ group. FC, fold change. VIP, variable important in projection.

## Data Availability

The raw dataset can be found in the MetaboLights database (www.ebi.ac.uk/metabolights/MTBLS3547, accessed on 24 March 2022).

## Supplementary Materials

The following supporting information can be downloaded at: https://www.mdpi.com/article/10.3390/foods11091286/s1, Table S1: Significantly differently expressed glycerophospholipids in the *MSTN*^+/−^ versus the WT pigs.
