# Supplementary material for: Myostatin Alteration in Pigs Enhances the Deposition of Long-Chain Unsaturated Fatty Acids in Subcutaneous Fat
Source: Foods. 2022 Apr 28;11(9):1286. doi: 10.3390/foods11091286 (PMC9105368; doi:10.3390/foods11091286)
Supplement: Supplementary file 1 [file foods-11-01286-s001.zip › Supplementary material.pdf]

Supplementary material

Table S1. Significantly differently expressed glycerophospholipids in the $MSTN^{+/-}$ versus the WT pigs.

MS2 Metabolites	WT	MSTN	FC (MSTN/WT)	t.test_p.value	VIP	regulated	MS2class
LysoPC 18:0	3633.707	9535.008531	2.6240	0.0004	2.5097	up	lysoPC
PC 32:0; PC (16:0/16:0)	13842.11	45363.48915	3.2772	0.0104	2.5115	up	PC
